# Multiple subacute bilateral cerebral infarcts in a 9-year-old Nigerian male: a case of childhood primary angiitis of the central nervous system

**DOI:** 10.11604/pamj.2018.31.99.13898

**Published:** 2018-10-10

**Authors:** Christian Chukwukere Ogoke, Ifeanyi Innocent Ike, Augustine Emeka Osuji, Kingsley Asinobi, Chukwudi Ernest Okeke

**Affiliations:** 1 Children’s Emergency Room (CHER), Ward 12 (children’s in-patient ward) and the Paediatric Neurology Clinic, Federal Medical Centre, Owerri, Nigeria

**Keywords:** Primary CNS vasculitis, cerebral infarcts, childhood PACNS, progressive limb weakness, aphasia

## Abstract

A Primary Angiitis of the Central Nervous System (PACNS) is ill-defined, complex and rare in children. Clinical presentation is variable, diagnosis is challenging and it is life-threatening but treatable. The index case is a 9-year old male who presented with progressively slurred speech, progressive weakness of the limbs, hemifacial weakness and vomiting. There were no clinical or laboratory features of systemic vasculitis. Neuroimaging showed multiple subacute infarcts in both cerebral hemispheres. He responded well to immunosuppressive therapy. The case report aims to underscore the need for high index of suspicion, early neuroimaging for progressive nonspecific decline in neurologic function and the treatable nature of the condition if diagnosis is made early.

## Introduction

Vasculitis refers to inflammation of blood vessels due to various causes [1]. It is called a Central Nervous System (CNS) vasculitis when the blood vessels of the brain and spinal cord are involved. CNS vasculitis is secondary if it complicates autoimmune disorders (e.g systemic lupus erythemaosis, dermatomyositis), viral or bacterial infections and systemic vasculitic disorders (e.g Wegener’s granulomatosis, polyarteritis nodosa, Takayasu’s arteritis, microscopic angiitis) [1, 2]. It occurs rarely without any associated systemic disorder or with little or no evidence of systemic inflammation and is called a primary angiitis of the CNS (PACNS) or primary CNS vasculitis. A primary angiitis of the CNS (PACNS) therefore refers to vascular inflammatory tissue injury confined to the CNS without any overt systemic process [2]. PACNS is uncommon in childhood. There are reported cases [3-6] in the literature but there are no reports from Nigeria. The diagnosis of PACNS is difficult because the clinical features are non-specific (no vasculitis-specific symptom or imaging sign) and there are no diagnostic criteria in children [1, 2]. However, the new international classification of childhood vasculitis [7] makes classification of childhood vasculitis possible without applying criteria used in adults. Many diagnostic measures (such as blood and cerebrospinal fluid tests, clinical chemistry, serology, neuroimaging) including a brain biopsy are required and they all have shortcomings [1, 2]. A brain biopsy, though rarely done, may provide unequivocal proof of vasculitis [1, 8]. The clinical course, severity and response to treatment also vary widely and thus this present case makes further contribution to medical knowledge.

## Patient and observation

The index patient is a 9-year old male who presented with complaints of progressively slurred speech, progressive weakness of right upper limb and both lower limbs and mouth deviation to the left side, of two weeks and vomiting of one hour - all prior to presentation. Onset of symptoms was insidious. Slurred speech progressed to aphasia and limb weakness progressed to inability to walk. No history of fever, catarrh, cough, leg swelling, difficulty in breathing, skin rash, haematuria, facial swelling, convulsions, headache, yellowness of the eyes, abdominal swelling, weight loss, night sweats or joint pains. No antecedent trauma to the head or ingestion of drugs or herbal preparations. His haemoglobin genotype was unknown. He presented to a private hospital where he was admitted and given subcutaneous low molecular weight heparin for two days on suspicion of stroke. His clinical state improved briefly and then deteriorated, necessitating referral to Federal Medical Centre, Owerri. General physical examination findings on admission were normal. Neurological examination showed a conscious child, cooperative with normal comprehension of speech but expressive aphasia, right sided facial weakness, no signs of meningeal irritation, normal muscle bulk, hypertonia in both upper and lower limbs, weakness of the limbs (power grade 3 on the Rt. sided limbs and grade 4- on the Lt. side), hyperreflexia bilaterally in both upper and lower limbs, absent abdominal reflexes, sustained ankle clonus and positive Babinski sign bilaterally. There were signs of incoordination (dysmetria, dysdiadochokinesia). Findings on examination of other systems were normal. Admitting diagnosis was encephalopathy 2oC encephalitis with differential diagnoses of space-occupying lesion (SOL) with raised intracranial pressure (RICP) and sickle cell CNS infarctive crisis.

Investigations done included: haemoglobin genotype, Full Blood Count (FBC), Erythrocyte Sedimentation Rate (ESR), Random Blood Sugar (RBS), Fasting Blood Sugar (FBS), blood film for Malaria Parasite (MP), Serum Electrolytes, Urea & Creatinine (SEUC), Magnetic Resonance Imaging (MRI) of the brain, Clotting profile with Activated Partial Thromboplastin Time (APTT). Results of investigations are shown in Table 1 and figures below.

**Table 1:** Laboratory investigations done and the results obtained

Test	Date	Result
HB Genotype	10/10/16	AA
RBS	10/10/16	108mg/dl (70-140mg/dl)
FBC	10/10/16	Hb 13.5g/dl (11-16g/dl), WBCT 14,800 (3-10.8 x 10^9^/L)Neu 84%(40-75%), Lym 16%(20-45%), platelets 345,000 (100-400 x 10^9^/L),blood film-normocytic,normochromic.No bands.
MP	10/10/16	Positive (+)
Urinalysis	10/10/16	amber &clear, PH 6.0,SG1.020,glucose,blood Protein,ketone,bilirubin, nitrite,ascorbic acid-all Negative, Urobil-normal .Microscopy: WBC,RBC,cast,crystals,yeast cell,epithelial cells-all absent
RBS	12/10/16	96mg/dl
Clotting profile	13/10/16	PT 12.9secs (control PT 16.1secs, INR 1.04), APTT 36.2secs (control 64.9secs), clotting time 5mins46secs (4-9mins), Bleeding time 2mins26secs (2-7mins), Fibrinogen 164.6(150-350mg/dl).
SEUC	13/10/16	Na^+^ 134 (135-150mmol/L), K^+^ 4.5 (3.5-5.0mmol/L), Cl^-^ 99(96-108mmo/L), Urea 19(15-40mg/dl), Cr 0.4(0.5-1.5mg/dl).
FBC	17/10/16	WBCT 9.2 x 10^9^/L (4-12 x 10^9^ /L), Neu 65% (50-70%) PLT479x10^9^/L(100-300), Lym 27% (20-60%),Eos 2%(1-5%), HB 13.3g/dl (12-16g/dl), MCV 86.3fL (80-100fL), MCH 30.2pg (27-34), MCHC 35g/dl (31-37),ESR 6mm/1hr (0-20mm/1hr) Westergreen. Blood film – normal

Hb -Haemoglobin, RBS-Random blood sugar, FBC- Full blood count, MP- blood film for malaria parasite, SG- specific gravity, SEUC- Serum electrolytes, urea & creatinine, Neu-neutrophils, Lym-lymphocytes, Eos-eosinophil,MCV-mean corpuscular volume, MCH-mean corpuscular haemoglobin, MCHC- mean corpuscular haemoglobin concentration, PLT-platelets, ESR-Erythrocyte sedimentation rate, PT- prothrombin time, APTT-Activated Partial Thromboplastin Time

**Figure 1:**
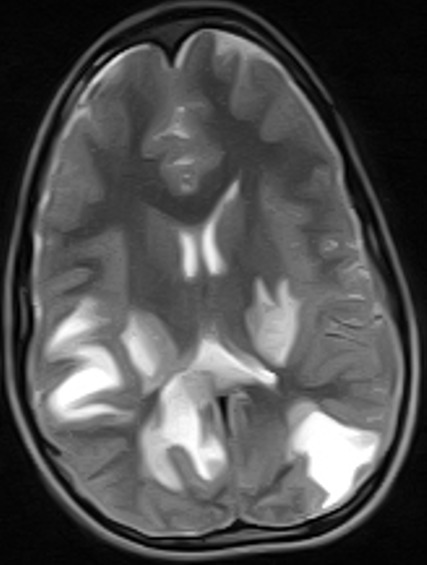
(MRI at presentation) is an axial T2 w sequence showing multifocal, irregular, hyperintense areas in the parietal and occipital regions

**Figure 2:**
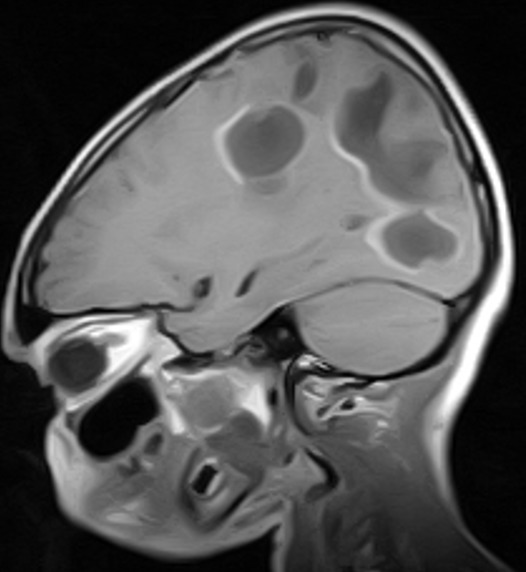
(MRI at presentation) is a post contrast T1 w parasagittal sequence showing multifocal, irregular hypointense areas with enhancing walls in the parieto-occipital region

**Figure 3:**
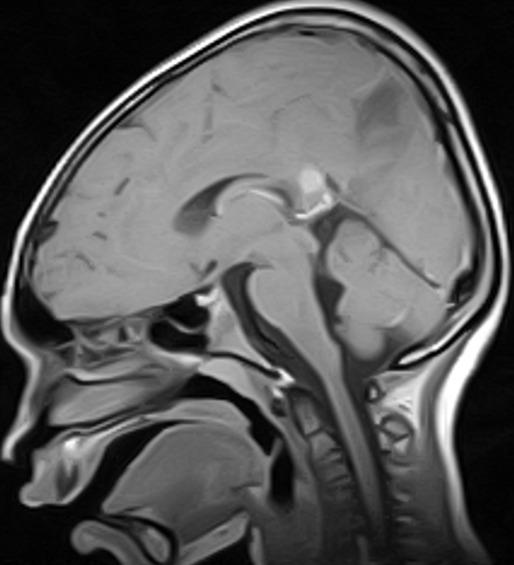
(MRI at presentation) is a T1 w midsagittal sequence showing an irregular hypointense area in the parietal region

The MRI brain showed multifocal areas of irregular abnormal signal intensities in the parietal and occipital regions suggestive of multiple subacute cerebral infarction (Figure 1, Figure 2, Figure 3). Following further review of patient’s clinical features, investigation results and MRI brain, the diagnosis was modified to Primary Angiitis of the CNS (PACNS). Due to vomiting on admission, patient was commenced initially on intravenous fluids for 24hrs and intravenous dexamethasone 4mg 6hrly for 48hrs. By the second day on admission, vomiting had subsided and patient commenced oral feeds and tabs prednisolone 20mg two times daily (1mg/kg/d). An intravenous antibiotic (Ceftriaxone 50mg/kg/d) was commenced while awaiting FBC result. An antiheliminthic (tab Albendazole 400mg stat) was administered. Physiotherapy was also instituted. Three days into admission, patient had become emotionally labile (cried easily when spoken to and was frustrated by his inability to talk) but general and neurological examination findings remained same. He received tabs Artemeter-Lumefantrin (480/80) due to a positive blood film for MP. By the seventh day on admission, remarkable improvement was noted. Patient’s speech had become audible though poorly fluent, and power had improved to grade 4^-^ on the right side similar to the left side. Gait was broad based and ataxic and he could not tandem walk. This improvement was steady and sustained. By the 4^th^ week on admission, power in all limbs had increased to grade 4^+^ but there was still subtle Rt. facial weakness, hyperreflexia, weakness of the hand muscles (Rt. > Lt.) with impaired fine motor function (handwriting, dressing). Signs of incoordination also persisted and increased appetite, weight gain of 6kg (35kg to 41kg) and “moon face” were noted. However, Blood Pressure (BP) (90/60mmHg) and vital signs (temperature, pulse rate, respiratory rate and oxygen saturation) remained normal.

Treatment was then modified with reduction of tabs prednisolone to 20mg on alternate days and addition of tabs cyclophosphamide 50mg on alternate days to synchronize with steroid-off days. Patient and his mother were counselled on the nature of the diagnosis, the possible prolonged therapy required, the possibility of residual deficits and the need for continued physiotherapy and occupational therapy. Due to the absence of trained occupational therapists in our locality, patient was provided with a small soft ball to squeeze frequently and advised to pick grains of rice from a tray repeatedly at home. He was to copy words, figures or diagrams from textbooks many times a day. All these were to improve his fine motor function especially handwriting. He was subsequently discharged and given weekly follow-up appointments in our neurology clinic. He was monitored for side effects of the immunosuppressive treatment with weekly FBC, ESR and BP monitoring and these remained normal all through the follow-up period.

**Figure 4:**
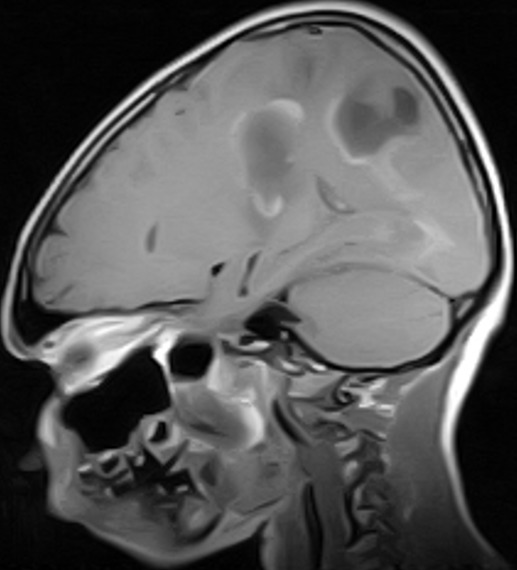
(MRI post treatment, 1yr after presentation) is a post contrast T1 w parasagittal sequence showing resolving multifocal, irregular hypointense areas with enhancing walls in the parietal region

**Figure 5:**
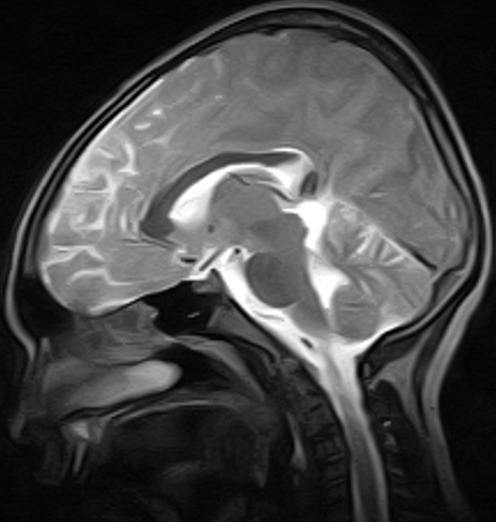
(MRI post treatment) is T1 w midsagittal sequence showing complete resolution of the lesion seen in the pretreatment midsagittal image

**Figure 6:**
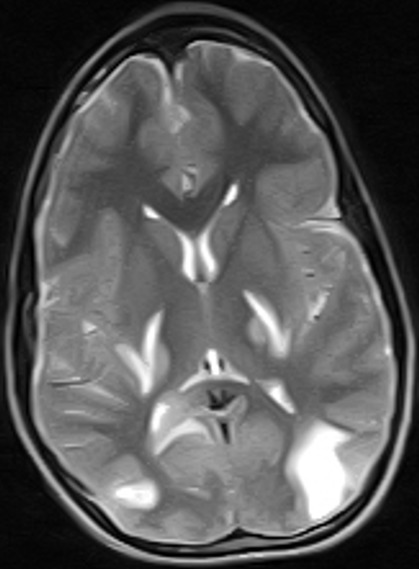
(MRI post treatment) is a T2 w axial sequence showing residual leisons

Subsequent follow-up visits (weeks 5-9) showed steady improvement with normal power returning to the limbs grossly, improving fine motor function (handwriting, performance of activities of daily living) and resolution of facial weakness, expressive aphasia and signs of upper motor neuron lesion. However, increased appetite, weight gain (41kg-53kg) and signs of incoordination remained. Further weekly tapering of prednisolone was done (10mg alternate days to 4mg alternate days to 2mg alternate days). By the 12^th^ week following diagnosis, hand grasp had become normal and handwriting legible and speed of writing good. Weight dropped to 42kg and he no longer had “moon face”. He resumed schooling with unimpaired participation. However, signs of incoordination such as dysmetria (past pointing) and impaired tandem walk persisted. Both drugs were finally stopped (after 3 months) and a repeat Magnetic Resonance Imaging (MRI) requested. Due to financial constraints and patient’s remarkable recovery, reimaging was delayed. Patient was seen again seven months later, about one year after admission and by then those signs of incoordination that persisted after discharge had all disappeared and patient was completely neurologically normal. A repeat brain MRI done at this time (one year after onset of illness) showed marked improvement with complete resolution of the some of the cortical lesions in the parietal and occipital regions bilaterally and residual hyperintensity in the periventricular and supraventricular cerebral white matter bilaterally involving the parieto-occipital regions predominantly; there were no new lesions (Figure 4, Figure 5, Figure 6).

## Discussion

The index case is the first reported case of childhood primary angiitis of the CNS (PACNS) from our centre and from Nigeria. The need for a high index of suspicion, the absence of vasculitis-specific symptoms or signs and the need for angiographic and or histologic evidence of vasculitis are diagnostic challenges. However, with increasing availability and use of neuroimaging (MRI) in Nigeria and scientific reports to increase awareness, more cases are likely to be diagnosed. The criteria characteristic of cPACNS [8] were present in our patient and the clinical features and course were in keeping with a monophasic, nonprogressive large/medium-vessel cPACNS (NPcPACNS) [8]. These features include: male sex, features of arterial ischaemic stroke, absence of seizures, the initial waxing and waning of symptoms prior to commencement of steroid therapy, normal blood inflammatory markers, bilateral cortical and subcortical infarction on brain MRI, resolution of symptoms with 12 weeks of immunosuppressive therapy and improved findings on reimaging with no new lesions.

An obvious omission in the evaluation of the index case is cerebrospinal fluid (csf) analysis. We omitted a lumbar puncture because at presentation the diagnosis was unclear and the presence of focal neurologic deficits and vomiting without fever suggested a Space Occupying Lesion (SOL) with raised intracranial pressure (RICP). Subsequently, with the marked response to immunosuppressive therapy, the caregiver declined consent. Nevertheless, csf analysis is an important laboratory test that is abnormal in < 50% of patients with large/medium-vessel cPACNS and > 90% of cases of small-vessel cPACNS [8].

At presentation, due to the unknown haemoglobin genotype of our patient, we also considered a diagnosis of CNS crisis of sickle cell anaemia (vasoocclusive crisis with CNS infarction). Sickle cell anaemia is a common cause of stroke in children in our environment. However, the absence of suggestive past medical and family history and the result of haemoglobin genotype (HbAA) quickly ruled it out. Another common differential diagnosis of PACNS is reversible cerebral vasoconstriction syndrome (RCVS); but our patient did not have any of the known precipitating factors (postpartum onset or vasoactive substances) or headache and he had an abnormal brain MRI; all features were against a diagnosis of RCVS [8].

The response of our patient to the immunosuppressive treatment given (prednisolone + tabs cyclophosphamide for 3 months) was amazing though he subsequently manifested side effects of steroid therapy. Neurologic recovery showed a steady progress with improvement in fine motor function (writing) enabling our patient to return to school. Surprisingly, signs of incoordination were still present beyond the time of discontinuation of immunosuppressive therapy. The signs of incoordination (cerebellar dysfunction) featured prominently in our patient and are not commonly emphasized in the cases reviewed in the literature.

Long term antithrombotic therapy (antiplatelet therapy such as aspirin) after discontinuation of immunosuppressive therapy is recommended especially in large/medium-vessel cPACNS because of increased risk for recurrent ischaemic events [8]. We omitted placing our patient on long term low dose aspirin after tailing off prednisolone. However, one could posit that this treatment is dispensable since our patient did very well without it. Nevertheless, the presence of other predisposing conditions to thrombosis should be sought for before considering exclusion of antiplatelet therapy.

## Conclusion

This case report intends to create more awareness and highlight the invaluable role of MRI in diagnosis and monitoring and the improving mortality with early diagnosis. Furthermore, the mainstay of treatment is immunosuppressive therapy and the signs of incoordination may persist up to a year before resolution in children with NPcPACNS.
